# Effects of Genetic and Production Type on Egg Cholesterol and the Yolk–Albumen Ratio in Slovenian Chicken Genotypes Under Standardised Conditions

**DOI:** 10.3390/ani15243588

**Published:** 2025-12-14

**Authors:** Dušan Terčič, Alenka Levart

**Affiliations:** Department of Animal Science, Biotechnical Faculty, University of Ljubljana, 1000 Ljubljana, Slovenia; alenka.levart@bf.uni-lj.si

**Keywords:** egg cholesterol, yolk-to-albumen ratio, chicken genotypes, production type, crossbred vs. purebred, standardised conditions

## Abstract

Eggs from ten Slovenian chicken genotypes—four layer-type purebreds, three meat-type purebreds, and three commercial F_1_ crossbreds—were analysed at 50 weeks of age (n = 118). All hens were reared indoors in identical floor-barn systems and fed the same complete layer diet, with feed intake restricted only in meat-type hens. Cholesterol was determined spectrophotometrically, and results included cholesterol per yolk dry matter and fresh yolk, per whole egg and egg content, total cholesterol per egg, and the yolk-to-albumen ratio. Production type significantly affected all traits (*p* < 0.0001): meat-type hens had the highest cholesterol concentrations and totals, layer-type purebreds were intermediate, and crossbreds the lowest. The yolk-to-albumen ratio showed the same gradient (≈0.44 in crossbreds, ≈0.46 in layer purebreds, ≈0.50 in meat types; *p* = 0.004), indicating that a larger yolk fraction contributes to higher total cholesterol. Reciprocal crossbreds did not differ in cholesterol levels (*p* > 0.05), suggesting negligible maternal or sex-linked effects. Under standardised conditions, genotype and production orientation were the main determinants of egg cholesterol and yolk proportion. These results provide reference values for Slovenian breeding and conservation populations and confirm exploitable genetic variability for future selection aimed at improving egg composition.

## Simple Summary

Egg cholesterol is found in the yolk and may vary according to the bird’s genetic background and production type. In this study, we compared ten Slovenian chicken genotypes: four layer-type purebreds, three meat-type purebreds, and three commercial F_1_ crossbreds. All hens were kept indoors under identical floor-barn conditions (same litter, lighting schedule, and diet; restricted feeding applied only for meat-type hens). Cholesterol concentrations were measured in yolk and whole eggs, and the yolk-to-albumen ratio was determined. A clear genetic gradient was observed: meat-type hens produced eggs with the highest cholesterol levels, layer-type purebreds were intermediate, and crossbreds had the lowest. The yolk-to-albumen ratio showed the same pattern, indicating that eggs with a relatively larger yolk contained more total cholesterol. Reciprocal crossbreds did not differ in cholesterol levels, suggesting minimal or no maternal or sex-linked effects. These results demonstrate that egg cholesterol levels are mainly determined by genotype and long-term selection history rather than short-term environmental factors. The study provides reference values for Slovenian conservation and breeding populations and suggests that genotype-related differences in yolk cholesterol concentration and yolk proportion contribute to natural variation in total egg cholesterol.

## 1. Introduction

Chicken eggs are nutrient-dense foods that provide high-quality protein, essential fatty acids, vitamins, and bioactive compounds, while naturally containing cholesterol concentrated in the yolk. Their compositional complexity has kept eggs at the centre of nutrition science and public debate. Recent meta-analyses and the updated Nordic Nutrition Recommendations [1] indicate that moderate egg consumption is compatible with cardiometabolic health; however, cohort studies continue to report inconsistent associations with cardiovascular outcomes [2,3,4,5,6,7].

These inconsistencies have renewed attention on the factors contributing to variability in egg composition—particularly yolk cholesterol—which is relevant to the characterisation and labelling of egg products. Yolk cholesterol deposition is physiologically well-understood [8,9]: oestrogens regulate hepatic synthesis of vitellogenins and yolk-targeted very-low-density lipoproteins (VLDLy), which transport lipids and cholesterol to developing follicles [9,10,11]. Several studies also indicate that long-term genetic selection pathways influence the regulation of these lipoprotein-mediated processes. Meat-type genotypes, selected primarily for rapid growth, are generally characterised by more lipogenic liver metabolism and increased hepatic lipid export [8,12,13], whereas layer-type genotypes selected for sustained egg production tend to exhibit more tightly regulated lipid allocation and differences in yolk precursor dynamics [10,11,14,15,16,17]. These physiological distinctions provide a mechanistic framework for understanding expected variation in yolk lipid and cholesterol deposition among divergent poultry genotypes.

Comparative studies across breeds suggest that egg composition reflects long-term selection history: local or heritage breeds generally produce eggs with a higher yolk fraction and greater cholesterol concentration than intensively selected commercial layers [18,19,20,21,22]. However, many earlier comparisons were confounded by differences in housing, feeding, or analytical methods, limiting interpretation of genetic effects. In particular, housing system and hen age are known to substantially affect egg weight and yolk cholesterol concentration [23,24], and numerous dietary interventions can also modify yolk cholesterol independently of genotype [25].

This study addresses this gap by providing the first integrated assessment of egg cholesterol variation across all Slovenian chicken genotypes maintained within national breeding programmes. Four layer-type and three meat-type purebreds, together with three commercial F_1_ crossbreds, were evaluated under identical housing, lighting, and dietary regimes, with eggs collected at a common physiological stage. The objectives were to (i) quantify yolk and whole-egg cholesterol across genotypes, (ii) quantify the main effects of production type (meat-type vs. layer-type) and genetic type (purebred vs. crossbred) on cholesterol traits as well as their interaction, and (iii) dissect potential maternal or sex-linked genetic effects by examining differences among breeds and between reciprocal crossbreds. By combining standardised management with validated analytical methods, this study establishes national reference values for egg cholesterol and provides new insight into genotype-related variation in egg composition among Slovenian poultry populations.

## 2. Materials and Methods

### 2.1. Chicken Genotypes, Rearing Conditions, and Egg Sampling

#### 2.1.1. Study Populations and Crossbred Formation

**Figure 1:**
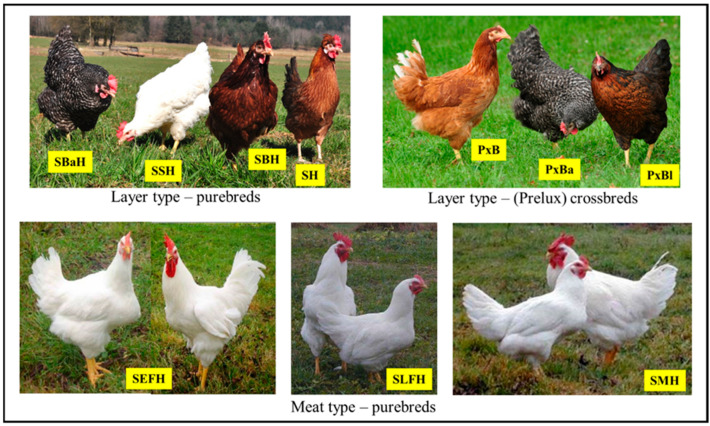
Chicken genotypes examined in the study, comprising four layer-type purebreds, three meat-type purebreds, and three commercial F_1_ crossbreds derived from layer-type purebreds.

The study included ten Slovenian chicken genotypes: four layer-type purebreds (Slovenian Brown Hen, SBH; Slovenian Silver Hen, SSH; Slovenian Barred Hen, SBaH; and Styrian Hen, SH), three meat-type purebreds (Slovenian Early Feathering Hen, SEFH; Slovenian Late Feathering Hen, SLFH; and Slovenian Meat Hen, SMH), and three commercial F_1_ crossbreds derived from the layer-type purebreds. The crossbreds—Prelux Brown (PxB), Prelux Barred (PxBa), and Prelux Black (PxBl)—were produced by SSH♂ × SBH♀, SBaH♂ × SBH♀, and SBH♂ × SBaH♀, respectively, and marketed under the Prelux trade name (Figure 1).

All purebred populations were maintained within long-term national breeding programmes coordinated by the Biotechnical Faculty, University of Ljubljana. Selection of Slovenian chicken breeds has been ongoing since the late 1960s, focusing on productive and adaptive traits. In layer-type purebreds, emphasis was placed on egg number and mass, whereas meat-type purebreds were selected for growth rate, with body weight at nine weeks as the main criterion. The Styrian Hen (SH), although selected for egg traits, was conserved as an indigenous population and was not used for crossbred formation.

#### 2.1.2. Husbandry, Housing, and Diet

All genotypes originated from nucleus flocks reared under identical conditions within the national gene-bank programme. Hens were housed indoors in barn systems with wood-shaving litter at a density of 5 hens per m^2^, provided with individual nests (1 per 5 hens), circular feeders (at least 4 cm space per hen), and nipple drinkers (no more than 10 hens per nipple). The lighting programme was gradually increased from 12 to 14 h of light per day and maintained throughout the laying period (approximately 15 lux at bird level). All flocks were preventively vaccinated against major poultry diseases, including Marek’s disease, Newcastle disease, infectious bronchitis, infectious bursal disease (Gumboro), *Salmonella* spp., egg drop syndrome, fowl pox, and infectious laryngotracheitis, in accordance with the official health-protection programme for pedigree and nucleus breeding flocks in Slovenia.

From 18 weeks of age, all hens received the same complete crumbled diet (16.2% crude protein, 11.3 MJ kg^−1^ ME, 3.1% ether extract, 3.0% crude fibre, 12.5% ash; Jata Emona Ltd., Ljubljana, Slovenia), containing 3.6% Ca, 0.45% P, 0.20% Na, 0.75% Lys, and 0.65% Met + Cys. The diet was a commercial corn–soybean meal–based complete layer diet; its declared nutrient composition (as-fed basis) is provided in Supplementary Table S1. All values represent calculated formulation data provided by the feed manufacturer. The same diet was used from 18 to 50 weeks of age in all layer-type hens and crossbreds; in meat-type hens, the same diet was fed restrictively as part of standard reproductive management. Individual feed intake was not measured because hens were housed in group pens. Fresh water was continuously available.

#### 2.1.3. Overview of Production and Egg Traits

Average body weight at 52 weeks showed clear differences among production types, ranging from approximately 1.9 to 2.6 kg in layer-type purebreds, 2.2 to 2.4 kg in layer-type crossbreds, and 3.4 to 4.2 kg in meat-type purebreds (Table 1). The onset of lay also varied: layer-type crossbreds reached 50% egg production at around 23 weeks of age, followed by layer-type purebreds at 25–26 weeks, while meat-type hens achieved this level considerably later, at 30–32 weeks (Table 1). Egg production up to 48 weeks reflected these genetic and physiological differences. Layer-type crossbreds showed the highest persistency, producing 170–177 eggs per hen, followed by layer-type purebreds with 104–173 eggs, whereas meat-type purebreds produced only 69–77 eggs during the same period. Despite pronounced differences in body size and laying performance, average egg mass showed partially overlapping ranges. Layer-type purebreds produced eggs weighing 46.4–61.1 g, crossbreds 57.2–63.2 g, and meat-type hens 59.6–64.9 g (Table 1). Notably, the Styrian Hen (SH) produced the smallest eggs among all genotypes, while the Slovenian Meat Hen (SMH) produced the heaviest. These differences in egg size and laying persistency contribute to the observed variation in total egg output among genotypes.

Detailed data on egg, albumen, and yolk traits of the ten Slovenian chicken purebreds and crossbreds are provided in Supplementary Table S2.

Egg sampling was standardised to minimise age-related variability and ensure physiological comparability among genotypes. At 50 weeks of age, corresponding to full maturity and stable laying performance, a random sample of 12 eggs per genotype (10 for SMH) was collected, ensuring that one freshly laid egg was obtained from each hen on the same morning to maintain independence of observations. All eggs were transported from the farm to the laboratory in an insulated cool box, stored at approximately 4 °C, and processed within 4–6 h of collection to minimise storage-related effects. Each egg was individually weighed and then separated into yolk and albumen for subsequent compositional and cholesterol analyses.

### 2.2. Cholesterol Extraction and Quantification

#### 2.2.1. Sample Preparation

Egg yolks were manually separated from the albumen, punctured, homogenised, and frozen at −20 °C prior to lyophilisation. Freeze-drying was performed for approximately 24 h. The dried samples were equilibrated to room temperature, weighed to the nearest 0.0001 g, homogenised using a laboratory grinder, vacuum-sealed, and stored at −20 °C until analysis. Lyophilised yolks were used for the determination of dry matter and cholesterol content. Dry matter was measured gravimetrically according to AOAC 925.30 [26].

#### 2.2.2. Extraction Procedure

For cholesterol determination, 0.25 g of lyophilised yolk was transferred into a 50 mL volumetric flask containing 1 g of quartz sand, 20 mL of 1.0 mol L^−1^ KOH in methanol, and 10 mL of isopropanol. The mixture was saponified under reflux at 67 °C for 30 min with continuous stirring, cooled to room temperature, and diluted to volume with isopropanol. After filtration, 1.0 mL of the filtrate was mixed with 0.6 mL of distilled water and 5 mL of hexane, vortexed, and centrifuged at 2500 rpm for 10 min. Two millilitres of the upper hexane phase were transferred to clean tubes using Gilson Pipetman P5000 manual air-displacement precision pipettes (Gilson Incorporated, Middleton, WI, USA) and evaporated to dryness under a stream of nitrogen at 50 °C. The residue was dissolved in 2 mL of 96% ethanol for spectrophotometric measurement.

#### 2.2.3. Colour Reagent Preparation

The ferric chloride colour reagent was prepared by dissolving 170 mg of FeCl_3_·6H_2_O in 4 mL of 80% H_3_PO_4_ in a 50 mL volumetric flask placed in an ice bath, followed by the slow addition of concentrated H_2_SO_4_ to the mark. The reagent was cooled to room temperature before use.

#### 2.2.4. Spectrophotometric Quantification

The analytical procedure was based on a modified Boehringer Mannheim colorimetric method [27] using FeCl_3_–H_3_PO_4_–H_2_SO_4_ as the chromogenic reagent. For each sample and standard, 2 mL of the ethanol extract was mixed with 2 mL of the colour reagent, vortexed, and incubated for 30 min at room temperature to allow full colour development. Absorbance was measured at 560 nm, corresponding to the wavelength of maximum absorbance (determined in the 450–800 nm range). Calibration was performed using five cholesterol standards (0–100 µg mL^−1^), yielding a linear relationship (A = 0.0098 × c + 0.0021; R^2^ = 0.998). To ensure that all samples remained within the linear range of the calibration curve, preliminary trials were conducted using varying volumes of the upper hexane phase obtained after saponification of egg yolk lipids across different chicken genotypes. Based on these trials, no additional dilution of samples was required prior to spectrophotometric analysis. The limits of detection (LOD) and quantification (LOQ), calculated as 3.3σ/slope and 10σ/slope, were 0.35 µg mL^−1^ and 1.06 µg mL^−1^, respectively [28]. Each sample was analysed in duplicate.

#### 2.2.5. Method Validation and Quality Control

Analytical precision was assessed through intra- and inter-day repeatability tests (n = 6 per day over three consecutive days), with coefficients of variation ranging from 2.3–4.8% (intra-day) and 3.5–6.1% (inter-day). Recovery experiments using cholesterol spikes or 5α-cholestane as an internal standard yielded recoveries of 96–103%. Method accuracy was verified using the certified reference material NIST SRM 1845a (Cholesterol in Whole Egg Powder) [29], with a certified value of 2.99 ± 0.10 mg g^−1^ DM; the measured value (3.05 ± 0.08 mg g^−1^) deviated by +2.0%.

### 2.3. Statistical Analyses

#### 2.3.1. Outcome Definitions

All statistical analyses were performed using SAS software version 9.4 [30]. Differences were considered statistically significant at *p* < 0.05. Cholesterol content in eggs was expressed in five ways: per gram of yolk dry matter (C_YDM), per gram of fresh yolk (C_YF), per gram of whole egg (C_Egg), per gram of total egg content (yolk plus albumen; C_Cont), and as the total amount of cholesterol in the entire egg (C_Total). In addition, the yolk-to-albumen ratio (YA_Ratio) was calculated as the ratio of yolk weight to albumen weight, both measured to the nearest 0.01 g. Five cholesterol traits capture complementary aspects of egg composition: yolk-based concentrations (C_YDM, C_YF) reflect metabolic regulation of yolk deposition, whole-egg and content-based concentrations (C_Egg, C_Cont) facilitate comparisons with food-composition studies, and total cholesterol per egg (C_Total) represents the absolute consumer-relevant load. The yolk-to-albumen ratio (YA_Ratio) provides structural context by describing relative yolk allocation.

#### 2.3.2. Data Screening and Transformations

Normality of residuals was assessed using the Shapiro–Wilk test and Q–Q plots (PROC UNIVARIATE), and homogeneity of variances using Levene’s test (PROC GLM). Three traits (C_YDM, C_YF, and YA_Ratio) satisfied normality (*p* > 0.05), whereas C_Egg, C_Cont, and C_Total deviated (*p* < 0.05). Logarithmic, square-root, and Box–Cox transformations were evaluated for the latter three traits. The Box–Cox transformation (PROC TRANSREG, geometric-mean scaling) successfully normalised C_Egg (λ = 0.55) and C_Cont (λ = 0.60). Analyses for these traits were therefore performed on the transformed scale. As no transformation achieved normality for C_Total, this trait was analysed using a rank-based general linear model (ranks computed prior to analysis) [31]. For this trait, differences between groups were expressed as Hodges–Lehmann estimates with 95% confidence intervals, representing robust nonparametric estimates of the median difference derived from ranked data. All parametric models (for C_YDM, C_YF, YA_Ratio, bc_C_Egg, and bc_C_Cont) were fitted on the analysis scale. Least-squares means (LS-means) and 95% confidence intervals were back-transformed to the original scale using the inverse-link option in SAS. Tables report back-transformed LS-means and their corresponding 95% confidence limits.

#### 2.3.3. Model Structure and Covariates

To assess the effects of production type, genetic type, and breed on each cholesterol trait and on egg morphological characteristics, a general linear model was fitted, as shown in Equation (1). Normally distributed and Box–Cox–transformed traits were analysed using general linear models (PROC GLM). For the rank-based model (C_Total), least-squares means were computed on ranked data, with yolk mass included as a covariate to maintain comparability with the parametric models (rank ANCOVA design). Heterogeneous variances were detected for C_YDM and C_YF (Levene’s *p* < 0.05); therefore, Welch’s correction was applied. Variances for other traits were homogeneous (*p* > 0.05). As each analysed egg originated from a different hen (one egg per hen), the experimental unit was the egg. All eggs were collected within a single facility under a common environment (uniform housing, lighting, and feeding). Thus, residual variance represents within-genotype variability. Nonetheless, potential genotype-level clustering (e.g., pen or breed-level effects) was considered when interpreting results, acknowledging that individual eggs within a genotype may share subtle non-independence due to shared management or genetic background. A separate GLM was fitted for each dependent variable to evaluate the effects of production type (layer-type vs. meat-type), genetic type (purebred vs. crossbred, nested within production type), and breed (nested within the combination of production and genetic types). Yolk mass was included as a covariate for C_Egg, C_Cont, and C_Total to adjust for its physiological influence on cholesterol deposition. For concentration-based traits (C_YDM, C_YF) and for YA_Ratio, yolk mass was excluded to avoid spurious correlations. Egg weight was not included as a covariate to prevent multicollinearity. Interaction terms between yolk mass and main effects were tested and found non-significant (*p* > 0.05); therefore, they were excluded from the final model. The general linear model was specified as:

Y_ijkl_ = μ + P_i_ + G_j_(P_i_) + B_k_(P_i_ × G_j_) + γ⋅YolkMass_(ijkl)_ + ε_ijkl_ (1)

where μ is the overall mean, P_i_ is the fixed effect of production type (i = 1, 2), G_j_(P_i_) is genetic type nested within production type (j = 1, 2), B_k_(P_i_ × G_j_) is breed nested within the combination of production and genetic types (k = 1, …, 10), γ is the regression coefficient for yolk mass (included when relevant), and ε_ijkl_ are residual errors associated with individual eggs. As meat-type genotypes included only purebreds, the genetic-type effect was estimated within the layer-type subset, while production-type and breed effects were evaluated across all genotypes.

#### 2.3.4. Sample Size and Power Considerations

Power calculations were initially based on the variability of cholesterol concentration in fresh yolk (C_YF), which in preliminary measurements showed a stable distribution and among the lowest within-genotype variance of all cholesterol traits. Using this trait as a representative endpoint, an a priori power analysis was performed in SAS (PROC POWER) for a two-sample t-test assuming α = 0.05 (two-sided) and β = 0.20 (80% power). With a standard deviation of approximately 1.3 mg g^−1^ and a total sample size of 118 eggs (about 12 per genotype), the design was expected to detect between-group differences of approximately 7–9% for production type (meat vs. layer) and 5–8% for genetic type within layers (purebred vs. crossbred). In the final dataset, the overall variability for C_YF was slightly higher (SD ≈ 2.3 mg g^−1^), whereas the within-group standard deviation remained close to 1.8 mg g^−1^. The observed mean contrasts were substantially larger than anticipated: about 2.9 mg g^−1^ between production types and 3.3 mg g^−1^ between genetic types within layers. When re-evaluated using these parameters, the corresponding effect sizes (Cohen’s d ≈ 1.5 and 3.0, respectively) yielded post hoc power estimates exceeding 0.99 for both contrasts. These results confirm that the experimental design provided more than sufficient statistical power to detect biologically meaningful differences in egg cholesterol traits under standardised environmental and analytical conditions.

### 2.4. Acknowledgement of AI Assistance

The authors acknowledge the use of ChatGPT-5 (OpenAI, San Francisco, CA, USA) during the preparation of this manuscript to assist with language editing and to generate the figures. All AI-generated content was critically reviewed and edited by the authors, who take full responsibility for the final version of the manuscript.

## 3. Results

### 3.1. Differences Between Layer-Type and Meat-Type Hens

Production type had a strong and significant influence on all cholesterol traits and on the yolk–albumen ratio (*p* < 0.0001 for all traits; Table 2, Supplementary Table S3).

Meat-type hens consistently produced eggs with higher cholesterol concentrations than layer-type hens. Cholesterol in egg content (C_Cont) averaged 5.10 mg g^−1^ in meat-type hens (95% CI: 4.92–5.29), compared with 4.07 mg g^−1^ in layer-type hens (95% CI: 3.97–4.17). A similar pattern was observed for yolk dry-matter cholesterol (C_YDM), which reached 31.06 mg g^−1^ DM in meat-type hens (95% CI: 30.25–31.88) and 25.28 mg g^−1^ DM in layer types (95% CI: 24.76–25.81). Total cholesterol per egg (C_Total) followed the same trend: median values were substantially higher in meat-type hens at 338.26 mg (IQR: 300.48–356.35) than in layer-type hens at 242.77 mg (IQR: 220.19–261.97). These results demonstrate that meat-type hens deposit both more cholesterol per unit of yolk dry matter and more total cholesterol per egg.

The structural composition of the egg also differed between production types. The yolk-to-albumen ratio (YA_Ratio) was higher in meat-type hens at 0.50 (95% CI: 0.49–0.51) than in layer-type hens at 0.45 (95% CI: 0.44–0.46) (*p* < 0.0001). This indicates that meat-type eggs contain a larger relative yolk fraction, which likely contributes to their greater total cholesterol content. Yolk mass, included as a covariate in the concentration- and content-based models, remained a significant positive predictor (*p* < 0.05), confirming that eggs with larger yolks contained proportionally more cholesterol irrespective of production type.

Two cholesterol traits—cholesterol in whole egg (C_Egg) and cholesterol in fresh yolk (C_YF)—are presented in Supplementary Table S3. Their patterns closely matched those of the traits retained in Table 2: meat-type hens exhibited higher values than layer-type hens (*p* < 0.0001), reinforcing the robustness of the production-type gradient across all cholesterol-related endpoints.

### 3.2. Genetic-Type Differences Within Production Type

Genetic type nested within production type significantly affected all cholesterol traits and the yolk–albumen ratio (*p* < 0.0001 for cholesterol traits; *p* = 0.004 for YA_Ratio; Table 2, Supplementary Table S3). A clear hierarchical pattern was observed—meat-type purebreds > layer-type purebreds > layer-type crossbreds. Crossbreds showed the lowest cholesterol levels across traits, with LS-means for C_Cont of 3.40 mg g^−1^ (95% CI: 3.26–3.54) and for C_YDM of 22.10 mg g^−1^ DM (95% CI: 21.31–22.89). Purebred layers exhibited intermediate values, with C_Cont of 4.74 mg g^−1^ (95% CI: 4.59–4.90) and C_YDM of 28.47 mg g^−1^ DM (95% CI: 27.78–29.15), whereas meat-type purebreds showed the highest cholesterol deposition, reaching 5.10 mg g^−1^ for C_Cont (95% CI: 4.92–5.29) and 31.06 mg g^−1^ DM for C_YDM (95% CI: 30.25–31.88). Total cholesterol per egg (C_Total) followed the same gradient, with median values ranging from 219.06 mg (IQR: 203.63–230.50) in crossbreds to 258.46 mg (IQR: 242.77–268.73) in purebred layers and 338.26 mg (IQR: 300.48–365.35) in meat-type hens.

The yolk-to-albumen ratio (YA_Ratio) reflected this structural progression, increasing from 0.44 (95% CI: 0.43–0.45) in crossbreds to 0.46 (95% CI: 0.45–0.47) in purebred layers and 0.50 (95% CI: 0.49–0.51) in meat-type hens (*p* = 0.004) (Table 2). Together, these trends indicate that genetic type determines cholesterol deposition through combined effects on yolk cholesterol concentration and relative yolk allocation.

The two cholesterol traits presented in Supplementary Table S3 (C_Egg and C_YF) showed the same genetic-type hierarchy—crossbreds < purebred layers < meat-type purebreds (*p* < 0.0001)—with LS-means and 95% CI fully consistent with the patterns described for the traits retained in Table 2.

Morphological characteristics of the same eggs supported the YA_Ratio pattern (Table 2). Yolk proportion increased from 26.95% (95% CI: 26.47–27.45) in crossbreds to 27.91% (95% CI: 27.49–28.34) in purebred layers and 29.49% (95% CI: 28.99–30.00) in meat-type hens (*p* = 0.0045), while albumen proportion showed the opposite trend—highest in crossbreds at 61.28% (95% CI: 60.66–61.90) and lowest in meat-type hens at 59.26% (95% CI: 58.62–59.89) (*p* = 0.0224). These structural differences align directly with the increasing YA_Ratio across genetic types.

### 3.3. Breed-Dependent Differences Across Cholesterol Traits

**Figure 2:**
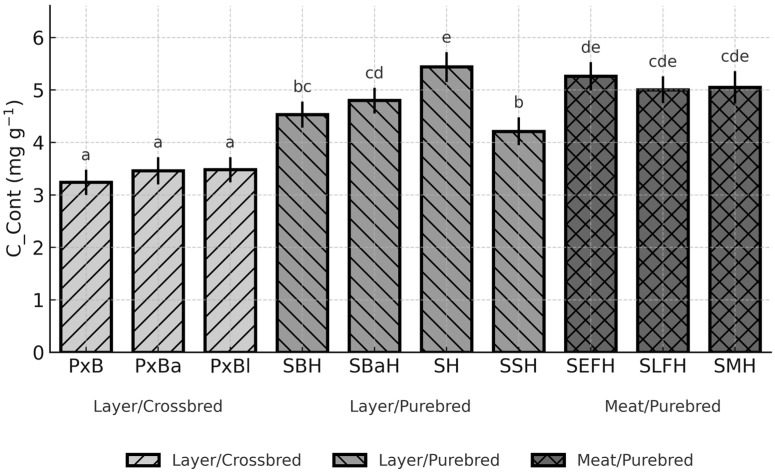
Breed-dependent variation in egg-content cholesterol concentration (C_Cont) across ten chicken genotypes (LSM, 95% CI; *p* < 0.0001). Different letters (a–e) above the bars indicate significant differences at *p* < 0.05.

**Figure 3:**
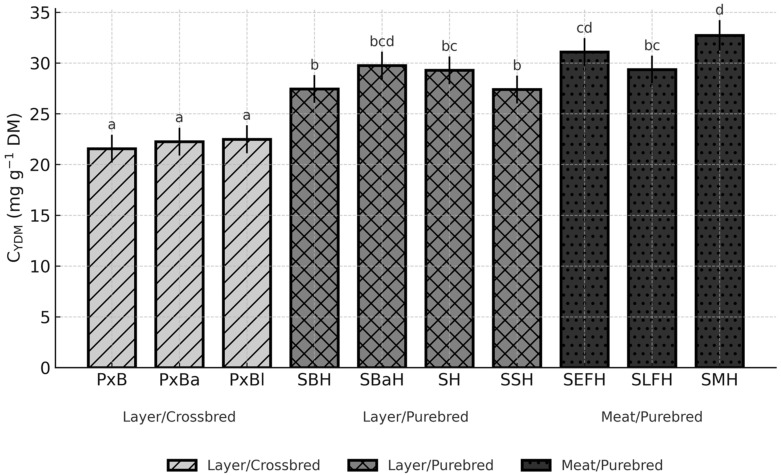
Breed-dependent variation in yolk cholesterol concentration (C_YDM) across ten chicken genotypes (LSM, 95% CI; *p* = 0.0056). Different letters (a–d) above the bars indicate significant differences at *p* < 0.05.

**Figure 4:**
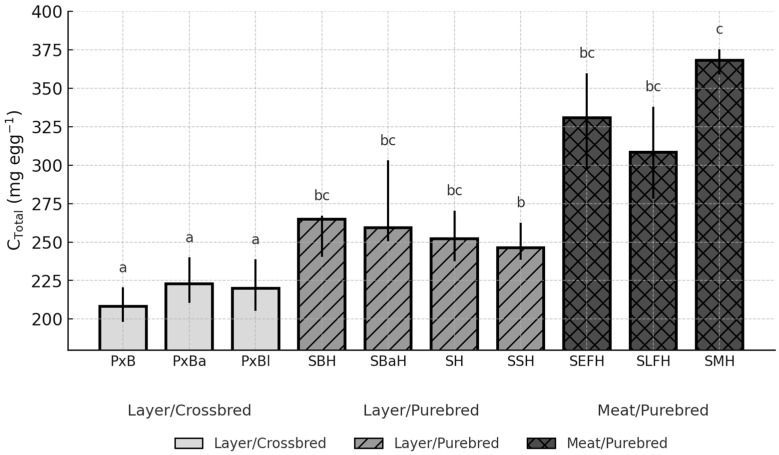
Breed-dependent variation in total egg cholesterol (C_Total) across ten chicken genotypes (Median, IQR; *p* = 0.1336). Different letters (a–c) above the bars indicate significant differences at *p* < 0.05.

Breed had a significant effect on egg-content cholesterol (C_Cont; *p* < 0.0001) and yolk cholesterol concentration (C_YDM; *p* = 0.0056), whereas the overall breed effect for total cholesterol per egg (C_Total) was not significant (*p* = 0.1336). To facilitate interpretation, the three cholesterol traits that best capture the key metabolic and nutritional dimensions of egg cholesterol (C_Cont, C_YDM, and C_Total) are presented graphically (Figure 2, Figure 3 and Figure 4). The remaining traits (C_YF and C_Egg), which are mathematically closely related to these primary measures, are summarised in Supplementary Table S3.

Breed-dependent differences in cholesterol concentration of egg content (C_Cont) were pronounced (Figure 2).

The lowest C_Cont occurred in PxB (3.24 mg g^−1^; 95% CI: 3.00–3.48), whereas the highest value was observed in the Styrian Hen, SH (5.44 mg g^−1^; CI: 5.15–5.72). This represents a significant contrast (*p* < 0.05) exceeding 2 mg g^−1^. Among the purebred layers, SBaH (4.80 mg g^−1^; CI: 4.55–5.04) and SBH (4.53 mg g^−1^; CI: 4.28–4.78) also differed significantly from the low-cholesterol crossbreds (*p* < 0.05) (Figure 2). High concentrations in SEFH (5.26 mg g^−1^; CI: 4.99–5.53) further emphasised the strong breed-related variation.

For C_YDM, breed differences were again significant (*p* < 0.05) (Figure 3).

The lowest concentration appeared in PxB (21.56 mg g^−1^ DM; CI: 20.19–22.93), while the highest was found in SMH (32.73 mg g^−1^ DM; CI: 31.23–34.23), a contrast of more than 11 mg g^−1^ DM (*p* < 0.05). Elevated values in SH (29.29 mg g^−1^ DM; CI: 27.92–30.65) and SBaH (29.75 mg g^−1^ DM; CI: 28.39–31.12) also significantly exceeded those of the crossbred genotypes (*p* < 0.05) (Figure 3).

Although C_Total did not differ significantly among breeds (*p* = 0.1336), the numerical contrasts were consistent (Figure 4).

PxB exhibited one of the lowest total cholesterol amounts (208.29 mg; CI: 198.06–220.54), whereas SMH reached by far the highest value (368.06 mg; CI: 358.60–375.27). The difference between these two extremes was nearly 160 mg per egg and statistically significant (*p* < 0.05). Variability among the layer-type purebreds was moderate, with SBH (264.65 mg; CI: 240.48–267.19) representing the upper range within this group (Figure 4).

Additional cholesterol traits (C_Egg, C_YF) presented in Supplementary Table S3 demonstrated similarly clear breed-dependent contrasts. Whole-egg cholesterol (C_Egg) ranged from PxB (2.85 mg g^−1^; CI: 2.64–3.06) to SH (4.85 mg g^−1^; CI: 4.60–5.10), both significantly different (*p* < 0.05). Fresh-yolk cholesterol (C_YF) spanned from PxB (10.87 mg g^−1^; CI: 10.17–11.56) to SMH (16.92 mg g^−1^; CI: 16.16–17.68) (*p* < 0.05) (Supplementary Table S3).

The yolk-to-albumen ratio (YA_Ratio) also varied significantly among breeds (*p* < 0.0001). SH, SEFH, and SLFH had the largest ratios (≈0.51–0.54), while SSH and the crossbreds had the smallest (≈0.40–0.48). Within the meat-type group, SMH displayed a distinctly (*p* < 0.05) lower YA_Ratio (~0.46) than SEFH (~0.53) and SLFH (~0.51), indicating intra–production-type heterogeneity in yolk allocation.

## 4. Discussion

This study provides the first comprehensive assessment of egg cholesterol content and distribution across all ten Slovenian chicken genotypes included in national breeding programmes. By standardising diet, housing, and physiological stage, we effectively isolated the genetic component of variation. The results revealed a clear and biologically consistent gradient: meat-type purebreds had the highest cholesterol levels, layer-type purebreds were intermediate, and commercial layer-type crossbreds had the lowest. This hierarchy demonstrates that egg cholesterol deposition is largely genetically determined and influenced by the historical breeding objectives and production orientation of the genotypes. Differences in the yolk-to-albumen ratio paralleled this gradient, indicating that egg structure plays an important role in the allocation and deposition of cholesterol.

### 4.1. Mechanistic Interpretation

The cholesterol gradient observed in our study aligns with established physiological mechanisms governing cholesterol metabolism during egg formation. Heavier, slow-laying hens typically exhibit a more lipogenic hepatic metabolism, characterised by enhanced synthesis of very-low-density lipoproteins (VLDLy) and vitellogenins under oestrogenic stimulation, which are the main carriers of cholesterol to developing follicles [8,9]. In contrast, modern layer lines selected for persistency and metabolic efficiency have been shown to downregulate hepatic cholesterol synthesis and reduce cholesterol transfer to the yolk [11,14,15].

In the present study, crossbred hens exhibited approximately 20–25% lower yolk cholesterol concentrations than their purebred parents, accompanied by a smaller yolk fraction (YA_Ratio ≈ 0.44 vs. 0.46). This difference likely reflects the cumulative effects of long-term selection for egg number and egg mass in the Slovenian layer population, which has been under systematic genetic improvement for more than five decades. Comparable patterns have been reported in other layer lines, where prolonged selection for laying persistency and feed efficiency has been associated with increased albumen synthesis and a proportionally smaller yolk fraction [32,33,34]. Our findings therefore support the interpretation that the lower egg cholesterol content in crossbreds primarily reflects a structural reallocation within the egg, characterised by a smaller yolk fraction and proportionally greater albumen mass. Structural processes, including genotype-specific follicle selection dynamics and reduced allocation of resources to the preovulatory yolk (lower YA_Ratio), limit the substrate available for cholesterol deposition [11,14,32,33,34]. In parallel, metabolic processes such as downregulated hepatic VLDLy and vitellogenin synthesis in high-performing layers reduce the flux of cholesterol-rich lipoproteins to developing follicles [8,9,14,15]. The combined effect of a smaller yolk mass and lower lipoprotein-mediated cholesterol delivery provides a mechanistic explanation for the production-type gradient observed in this study, with crossbred layers exhibiting the lowest, purebred layers intermediate, and meat-type hens the highest yolk cholesterol values.

### 4.2. Breed-Level Differences

After accounting for production type, significant differences among breeds remained. Rankings for C_YDM, C_YF, and YA_Ratio were broadly consistent, confirming that both yolk cholesterol concentration and yolk proportion contribute to breed-specific profiles. The Slovenian Meat Hen (SMH) had the highest yolk cholesterol concentration but only an intermediate yolk-to-albumen ratio (~0.46). Consequently, its total cholesterol per egg was comparable to that of the Styrian Hen (SH) and the Slovenian Early Feathering Hen (SEFH), which had lower yolk cholesterol concentrations but relatively larger yolk fractions. These findings indicate that similar total egg cholesterol levels can arise through different structural–metabolic combinations—either a more cholesterol-dense yolk or a proportionally larger yolk volume. A plausible explanation for these breed-specific patterns is that yolk cholesterol concentration and yolk size are regulated by partially independent physiological pathways. Breeds such as the SMH may exhibit a more intensive hepatic lipogenic metabolism—including higher synthesis and export of VLDLy and vitellogenins under oestrogenic stimulation—which increases cholesterol deposition per unit of yolk mass [8,9,10,11,14,15,16,17]. In contrast, breeds like SH and SEFH, which show lower yolk cholesterol density but larger yolk fractions, may differ in follicular growth dynamics and in the efficiency of yolk precursor uptake via receptor-mediated endocytosis. Such differences likely reflect breed-specific variation in oestrogen sensitivity, rates of lipoprotein assembly, and the regulation of key genes involved in yolk precursor synthesis and transport (e.g., *APOB*, *MTTP*, *VLDLR*, *LPL*) [16,17,35,36]. Consequently, similar total egg cholesterol can arise either through enhanced metabolic deposition into a smaller yolk or through structural allocation into a larger yolk volume.

Compared with other European studies, the cholesterol concentrations measured in Slovenian layer-type purebreds (≈27–30 mg g^−1^ DM) are similar to values reported for Italian dual-purpose breeds—Pepoi at approximately 28 mg g^−1^ DM and Robusta around 25 mg g^−1^ DM [18] and correspond with data for native Polish lines (27.6–28.0 mg g^−1^ DM) [23] and Czech datasets ranging from 23.7 to 31.0 mg g^−1^ DM, depending on hen age and system [24,37]. These patterns indicate that Slovenian purebreds share compositional characteristics with traditional dual-purpose or conserved populations. Furthermore, studies comparing local breeds with modern hybrids show lower yolk cholesterol per egg in hybrids (e.g., 219 vs. 258 mg/yolk) [38], consistent with long-term selection in commercial lines for production traits.

Among Slovenian breeds, the positive association between yolk cholesterol concentration (C_YDM) and the yolk-to-albumen ratio (YA_Ratio) suggests that part of the breed-specific variation in total egg cholesterol may be explained by differences in egg structure. Breeds with a relatively larger yolk fraction tended to have higher yolk cholesterol concentrations, consistent with greater yolk lipid deposition. However, these structural effects are unlikely to be the sole explanation. As discussed above, modern high-performing layers generally exhibit reduced hepatic cholesterol synthesis and modified lipoprotein-mediated uptake, while transcriptomic evidence indicates breed-dependent regulation of key genes involved in yolk precursor synthesis and transport (e.g., *APOB*, *MTTP*, *VLDLR*, *FASN*) [16,17,35]. Overall, both structural and metabolic factors, shaped by long-term selection history, contribute to the diversity of yolk cholesterol profiles among Slovenian chicken breeds.

### 4.3. Reciprocal Crosses and Inheritance

Reciprocal comparisons between PxBl (SBH♂ × SBaH♀) and PxBa (SBaH♂ × SBH♀) crossbreds revealed no significant differences in any cholesterol-related trait. Although the reciprocal comparison revealed no statistically detectable maternal or sex-linked differences in any cholesterol-related trait, the possibility of small effects cannot be excluded given the sample size and the specific cross combinations analysed. The experimental design does not permit estimation of heritability or separation of additive genetic and common-environmental influences; therefore, breeding implications should be interpreted with caution. It should be emphasised that, although clear phenotypic differences between genotypes were observed, our study does not provide estimates of heritability or genetic variance for yolk cholesterol and thus the observed genotype-associated patterns should not be interpreted as direct evidence of inheritance or of unrestricted selection potential, especially given that endocrine and metabolic regulation imposes biological limits on how far yolk cholesterol deposition can be reduced.

Previous studies have documented moderate heritability and consistent selection responses for yolk cholesterol [39,40], although correlated changes in egg weight and hatchability have also been reported. These findings collectively suggest that genetic improvement is feasible but may influence multiple reproductive traits simultaneously, which warrants careful consideration in breeding objectives.

### 4.4. Physiological and Evolutionary Context

Yolk cholesterol is essential for embryonic membrane formation and steroidogenesis, as it provides structural lipids and precursors for steroid hormones during early embryogenesis [9,41,42]. Its concentration is tightly regulated by hepatic lipoprotein synthesis and receptor-mediated uptake into the oocyte [9]. The range observed in this study (≈21–33 mg g^−1^ DM) corresponds well with previously reported physiological limits for hens of similar age. For example, studies have shown that yolk cholesterol concentrations vary by strain and age, albeit often at somewhat lower absolute values, emphasising that deposition is under physiological control [37]. The relatively narrow variation among Slovenian genotypes suggests that natural and artificial selection may help maintain cholesterol deposition within physiologically optimal limits, consistent with reports of moderate heritability and evidence of stabilising selection for yolk cholesterol [15].

At the same time, the structured distribution of cholesterol values across genotypes provides insight into broader reproductive allocation patterns. Meat-type hens, which exhibited the highest yolk cholesterol concentrations together with a relatively large yolk fraction, appear to invest more nutrients and sterols in each individual egg—an allocation pattern consistent with their slower laying rhythm and greater body mass [42,43,44]. In contrast, the commercial crossbreds, which showed lower yolk cholesterol concentrations and smaller yolk proportions, achieve high annual egg numbers with comparatively lower per-egg investment, reflecting long-term selection for metabolic efficiency and laying persistency [18,19,20,21,22,32,33,34]. These contrasting allocation patterns align with established life-history principles describing how organisms balance investment in the size and provisioning of individual offspring with overall reproductive output [43,44]. In this context, the genotype-dependent cholesterol gradient observed here offers a coherent physiological and evolutionary explanation for differences in yolk deposition strategies among Slovenian chicken genotypes.

### 4.5. Methodological Considerations

A major strength of this study lies in its controlled experimental design. All genotypes were reared under identical housing, lighting, and dietary conditions, and eggs were collected at a uniform physiological stage (50 weeks). Analytical reliability was ensured through the use of certified reference materials and duplicate assays, providing high confidence that the observed differences reflect genuine genotypic variation rather than environmental effects.

Several limitations should also be acknowledged. The study was conducted at a single age point; therefore, longitudinal sampling would be required to assess age-related changes in yolk cholesterol metabolism [45,46]. Feeding regimens differed slightly between production types, as meat-type hens were subjected to restricted feeding to control body weight. However, this represents standard management practice for heavy genotypes [13,47] rather than an experimental artefact and accurately reflects real production conditions. Finally, focusing solely on cholesterol provides only a partial view of egg lipid metabolism. Future studies integrating phospholipid and fatty acid profiling would yield a more comprehensive understanding of yolk composition [48,49].

### 4.6. Breeding and Practical Implications

Crossbred hens in this study produced eggs with markedly lower yolk and total cholesterol than their purebred counterparts, demonstrating clear genetic differentiation among genotypes. Together with previous evidence of moderate heritability for yolk cholesterol [15], these findings support the view that egg cholesterol is a genetically determined and potentially selectable trait. Although cholesterol has not been an explicit breeding objective in Slovenian programmes, the results indicate genetic potential for further improvement. Integrating compositional traits such as yolk cholesterol concentration and the yolk-to-albumen ratio into multi-trait genomic evaluations (e.g., GBLUP or ssGBLUP) could enable balanced progress between production efficiency and nutritional quality [50,51,52].

Crossbred eggs contained approximately one quarter less total cholesterol than eggs from purebred and meat-type hens, illustrating substantial genotype-related variation in this trait. These genotype-specific values contribute to the understanding of natural variability in egg composition and may support compositional characterisation and the development of nutritional-component databases [53,54]. Interpretation of these results beyond their compositional and genetic context should be made with caution, as they were obtained under controlled experimental conditions.

### 4.7. Future Directions

Future research should prioritise (i) multi-omics analyses (genomic, transcriptomic, lipidomic) to identify regulatory pathways controlling yolk lipid deposition [9,16,17,35,48,49], (ii) longitudinal studies across the laying cycle to capture age-related changes in yolk precursor synthesis and egg composition [14,15,45,46], and (iii) breeding validation through controlled selection and genomic-evaluation experiments targeting yolk cholesterol concentration and the yolk–albumen ratio [15,38,39,40,41,50,51,52]. Integrating genomic, molecular, and lipidomic approaches will enable a more comprehensive understanding of yolk lipid metabolism and may support breeding decisions and future research strategies.

The genotype-dependent differences observed in this study offer a valuable empirical foundation for linking phenotypic variation in yolk cholesterol and yolk structure with underlying genetic and metabolic determinants. Previous work has shown that traits related to yolk lipid deposition are influenced by heritable variation in hepatic cholesterol synthesis, lipoprotein export and oocyte uptake pathways [8,9,10,11,14,15,16,17,35]. By providing clearly differentiated lipid phenotypes across production and genetic types under fully standardised conditions, our findings establish a relevant model for future molecular studies aimed at elucidating the genetic basis of nutritionally important egg traits. Such integrative approaches are important for clarifying the genetic basis of variation in yolk lipid traits and for supporting breeding strategies based on genetic information.

## 5. Conclusions

Under standardised housing, diet, and lighting, genotype and production orientation were identified as the primary determinants of egg cholesterol in Slovenian chicken genotypes. Meat-type purebreds consistently exhibited the highest cholesterol levels, purebred layers intermediate values, and commercial F_1_ crossbreds the lowest, for both concentration- and content-based traits. The yolk-to-albumen ratio followed the same pattern (crossbreds < purebred layers < meat types), indicating that egg structural composition—particularly the relative yolk fraction—contributes substantially to total cholesterol content in addition to metabolic factors. Reciprocal crosses showed no evidence of maternal or sex-linked effects, supporting a predominantly additive genetic basis of variation.

The study establishes reference values for cholesterol and yolk proportion traits specific to Slovenian chicken genotypes maintained in conservation and breeding programmes under uniform experimental conditions. These data provide a standardised baseline for comparative and genetic studies but should not be interpreted as national composition tables applicable to all production systems. Extrapolation to field or free-range conditions requires caution, as environmental factors such as diet, housing, or hen age can alter lipid metabolism and egg composition.

Within these defined limits, the findings demonstrate that genetic selection—by favouring lines with lower yolk cholesterol and/or reduced yolk-to-albumen ratio—offers a sustainable, feed-independent approach to producing eggs with naturally reduced cholesterol. However, the Slovenian Meat Hen illustrates that selection targeting structural traits alone may not reliably reduce total egg cholesterol, because exceptionally high yolk cholesterol concentration can offset reductions in yolk proportion. This exception underscores that genotype-specific cholesterol outcomes depend jointly on yolk density and yolk proportion and that multiple structural and metabolic selection pathways may be required to achieve eggs with genuinely lower intrinsic cholesterol content. Taken together, these results indicate that selection based solely on yolk proportion may not achieve a reduction in total cholesterol; as illustrated by the Slovenian Meat Hen, yolk cholesterol concentration must also be considered, so structural and metabolic traits should be integrated in breeding.

## Figures and Tables

**Table 1:** Production and egg traits of ten Slovenian chicken genotypes between 24 and 48 weeks of age under standardised conditions.

Production Type/Genetic Type	Purebred/Crossbred	Total Hens Housed (n)	Age at 50% Egg Production (Weeks)	Average Body Weight (g ± SD) at 52 Weeks	Eggs per Live Hen to 48 Weeks ^1^	Average Egg Mass (g ± SD) to 48 Weeks ^2^
Layer-type purebreds	Slovenian Brown Hen (SBH)	162	25	2042.4 ± 169.9	172.8	61.13 ± 6.36
Layer-type purebreds	Slovenian Silver Hen (SSH)	162	26	2272.2 ± 290.0	167.6	59.86 ± 5.58
Layer-type purebreds	Slovenian Barred Hen (SBaH)	162	26	2613.6 ± 247.4	134.4	56.55 ± 5.42
Layer-type purebreds	Styrian Hen (SH)	102	26	1916.1 ± 219.6	104.0	46.37 ± 4.91
Layer-type crossbreds	Prelux Brown (PxB)	114	23	2169.5 ± 193.1	171.2	63.18 ± 5.83
Layer-type crossbreds	Prelux Barred (PxBa)	113	23	2388.1 ± 216.3	170.4	57.17 ± 5.63
Layer-type crossbreds	Prelux Black (PxBl)	115	23	2351.1 ± 196.9	176.8	61.36 ± 6.11
Meat-type purebreds	Slovenian Early Feathering Hen (SEFH)	320	32	3868.0 ± 406.1	70.5	61.73 ± 4.19
Meat-type purebreds	Slovenian Late Feathering Hen (SLFH)	323	31	3371.2 ± 397.7	77.4	59.51 ± 3.82
Meat-type purebreds	Slovenian Meat Hen (SMH)	326	30	4188.0 ± 494.1	68.8	64.90 ± 3.15

^1^ Eggs per live hen were calculated as total eggs produced per live hen to 48 weeks of age. ^2^ A random sample of 30 eggs per genotype was collected and weighed at four-week intervals. In layer-type breeds, sampling was conducted from 24 to 48 weeks of age (seven sampling points), whereas in meat-type breeds it was performed from 28 to 48 weeks of age (six sampling points).

**Table 2:** Effects of production type and genetic type on egg cholesterol traits, yolk-to-albumen ratio, and selected egg morphological characteristics in Slovenian chicken genotypes.

(**A**) **Cholesterol Traits: Effect of Production Type**				
**Trait**	**Layer Type**	**Meat Type**		***p*-Value**
Cholesterol in egg content—C_Cont (mg g^−1^) ^1^	4.07 ^a^ (3.97–4.17)	5.10 ^b^ (4.92–5.29)		*p* < 0.0001
Cholesterol in yolk dry matter—C_YDM (mg g^−1^ DM) ^1^	25.28 ^a^ (24.76–25.81)	31.06 ^b^ (30.25–31.88)		*p* < 0.0001
Total cholesterol per egg—C_Total (mg egg^−1^) ^2^	242.77 ^a^ (220.19–261.97)	338.26 ^b^ (300.48–356.35)		*p* < 0.0001
Yolk-to-albumen (YA) ratio	0.45 ^a^ (0.44–0.46)	0.50 ^b^ (0.49–0.51)		*p* < 0.0001
(**B**) **Cholesterol Traits: Effect of Genetic Type Nested Within Production Type**				
**Trait**	**Crossbred—Layer**	**Purebred—Layer**	**Purebred—Meat**	***p*-Value**
Cholesterol in egg content—C_Cont (mg g^−1^) ^1^	3.40 ^a^ (3.26–3.54)	4.74 ^b^ (4.59–4.90)	5.10 ^c^ (4.92–5.29)	*p* < 0.0001
Cholesterol in yolk dry matter—C_YDM (mg g^−1^ DM) ^1^	22.10 ^a^ (21.31–22.89)	28.47 ^b^ (27.78–29.15)	31.06 ^c^ (30.25–31.88)	*p* < 0.0001
Total cholesterol per egg—C_Total (mg egg^−1^) ^2^	219.06 ^a^ (203.63–230.50)	258.46 ^b^ (242.77–268.73)	338.26 ^c^ (300.48–365.35)	*p* < 0.0001
Yolk to albumen (YA) ratio	0.44 ^a^ (0.43–0.45)	0.46 ^b^ (0.45–0.47)	0.50 ^c^ (0.49–0.51)	*p* = 0.0040
(**C**) **Egg Morphological Characteristics: Effect of Genetic Type Nested Within Production Type**				
**Trait**	**Crossbred—Layer**	**Purebred—Layer**	**Purebred—Meat**	***p*-Value**
Egg mass (g) ^1^	72.90 ^a^ (71.99–73.82)	64.89 ^b^ (64.11–65.68)	71.11 ^c^ (70.17–72.06)	*p* < 0.0001
Yolk proportion (%) ^1^	26.95 ^a^ (26.47–27.45)	27.91 ^b^ (27.49–28.34)	29.49 ^c^ (28.99–30.00)	*p* = 0.0045
Albumen proportion (%) ^1^	61.28 ^a^ (60.66–61.90)	60.32 ^a^ (59.78–60.85)	59.26 ^b^ (58.62–59.89)	*p* = 0.0224

^1^ LS-means (95% CI) from the parametric GLM. ^2^ Medians (IQR) from the rank-based ANCOVA. Different superscript letters (a, b, c) within a row indicate significant differences between production types (A) and between genetic types nested within production types (B,C) (*p* < 0.05; Tukey–Kramer test applied to raw or ranked data, as appropriate).

## Data Availability

All data supporting the findings of this study, as well as the SAS code used for the statistical analyses, are available from the corresponding author upon reasonable request.

## Supplementary Materials

The following supporting information can be downloaded at: https://www.mdpi.com/article/10.3390/ani15243588/s1, Table S1: Nutrient composition, ingredient list, and additives of the complete corn–soybean meal–based diet; Table S2: Average egg, albumen, and yolk masses, proportions, and yolk-to-albumen ratio in eggs from purebred and crossbred hens; Table S3: Cholesterol traits (C_Egg, C_YF) in eggs of Slovenian chicken genotypes under standardised conditions (LS-means ± 95% CI).

## Abbreviations

The following abbreviations are used in this manuscript:

AOAC	Association of Official Analytical Chemists
*APOB*	Apolipoprotein B
BC	Box–Cox (transformation)
C_Cont	Cholesterol concentration in egg content
C_Egg	Cholesterol concentration in whole egg
C_Total	Total cholesterol per egg
C_YDM	Cholesterol concentration in yolk dry matter
C_YF	Cholesterol concentration in fresh yolk
DM	Dry matter
EU	European Union
FeCl_3_	Ferric chloride
F_1_	First filial generation (crossbred generation)
GBLUP	Genomic best linear unbiased prediction
GLM	General linear model
*HMGCR*	3-Hydroxy-3-methylglutaryl-CoA reductase
LOD	Limit of detection
LOQ	Limit of quantification
*LPL*	Lipoprotein lipase
LS-mean	Least-squares mean
Met + Cys	Methionine plus cysteine
*MTTP*	Microsomal triglyceride transfer protein
NIST	National Institute of Standards and Technology
PxB	Prelux Brown
PxBa	Prelux Barred
PxBl	Prelux Black
SBH	Slovenian Brown Hen
SBaH	Slovenian Barred Hen
SEFH	Slovenian Early Feathering Hen
SH	Styrian Hen
SLFH	Slovenian Late Feathering Hen
SMH	Slovenian Meat Hen
SSH	Slovenian Silver Hen
SSG	Standardised sampling group
*VLDL*	Very-low-density lipoprotein
VLDLy	Yolk-targeted very-low-density lipoprotein
wk	Week(s)
YA_Ratio	Yolk-to-albumen ratio

## References

[1] Nordic Council of Ministers (2023). Nordic Nutrition Recommendations 2023: Towards Sustainable and Healthy Diets.

[2] Rouhani M.H., Rashidi-Pourfard N., Salehi-Abargouei A., Karimi M., Haghighatdoost F. (2018). Effects of Egg Consumption on Blood Lipids: A Systematic Review and Meta-Analysis of Randomized Clinical Trials. J. Am. Coll. Nutr..

[3] Drouin-Chartier J.-P., Chen S., Li Y., Schwab A.L., Stampfer M.J., Sacks F.M., Rosner B., Willett W.C., Hu F.B., Bhupathiraju S.N. (2020). Egg Consumption and Risk of Cardiovascular Disease: Three Large Prospective US Cohort Studies, Systematic Review, and Updated Meta-Analysis. BMJ.

[4] Carter S., Connole E.S., Hill A.M., Buckley J.D., Coates A.M. (2023). Eggs and Cardiovascular Disease Risk: An Update of Recent Evidence. Curr. Atheroscler. Rep..

[5] Virtanen J.K., Larsson S.C. (2024). Eggs—A Scoping Review for Nordic Nutrition Recommendations 2023. Food Nutr. Res..

[6] Li M.-Y., Chen J.-H., Chen C., Kang Y.-N. (2020). Association between Egg Consumption and Cholesterol Concentration: A Systematic Review and Meta-Analysis of Randomized Controlled Trials. Nutrients.

[7] Zhao B., Gan L., Graubard B.I., Männistö S., Albanes D., Huang J. (2022). Associations of Dietary Cholesterol, Serum Cholesterol, and Egg Consumption with Overall and Cause-Specific Mortality: Systematic Review and Updated Meta-Analysis. Circulation.

[8] Hermier D. (1997). Lipoprotein Metabolism and Fattening in Poultry. J. Nutr..

[9] Walzem R.L., Hansen R.J., Williams D.L., Hamilton R.L. (1999). Estrogen Induction of VLDLy Assembly in Egg-Laying Hens. J. Nutr..

[10] Nimpf J., Schneider W.J. (1991). Receptor-Mediated Lipoprotein Transport in Laying Hens. J. Nutr..

[11] Song X., Wang D., Zhou Y., Sun Y., Ao X., Hao R., Gao M., Xu Y., Li P., Jia C. (2023). Yolk Precursor Synthesis and Deposition in Hierarchical Follicles and Effect on Egg Production Performance of Hens. Poult. Sci..

[12] Nangsuay A., Molenaar R., Meijerhof R., van den Anker I., Heetkamp M.J.W., Kemp B., van den Brand H. (2015). Differences in Egg Nutrient Availability, Development, and Nutrient Metabolism of Broiler and Layer Embryos. Poult. Sci..

[13] Carney V.L., Anthony N.B., Robinson F.E., Reimer B.L., Korver D.R., Zuidhof M.J., Afrouziyeh M. (2022). Evolution of Maternal Feed Restriction Practices over 60 Years of Selection for Broiler Productivity. Poult. Sci..

[14] Liu X.-T., Lin X., Mi Y.-L., Zeng W.-D., Zhang C.-Q. (2018). Age-Related Changes of Yolk Precursor Formation in the Liver of Laying Hens. J. Zhejiang Univ. Sci. B.

[15] Chen X., Zhu W., Du Y., Liu X., Geng Z. (2019). Genetic Parameters for Yolk Cholesterol and Transcriptional Evidence Indicate a Role of Lipoprotein Lipase in the Cholesterol Metabolism of the Chinese Wenchang Chicken. Front. Genet..

[16] Li H., Wang T., Xu C., Wang D., Ren J., Li Y., Tian Y., Wang Y., Jiao Y., Kang X. (2015). Transcriptome Profile of Liver at Different Physiological Stages Reveals Potential Mode for Lipid Metabolism in Laying Hens. BMC Genom..

[17] Cui Z., Ning Z., Deng X., Du X., Amevor F.K., Liu L., Kang X., Tian Y., Wang Y., Li D. (2022). Integrated Proteomic and Metabolomic Analyses of Chicken Ovary Revealed the Crucial Role of Lipoprotein Lipase on Lipid Metabolism and Steroidogenesis during Sexual Maturity. Front. Physiol..

[18] Rizzi C., Chiericato G.M. (2010). Chemical Composition of Meat and Egg Yolk of Hybrid and Italian Breed Hens Reared Using an Organic Production System. Poult. Sci..

[19] Lordelo M., Cid J., Cordovil C.M.S., Alves S.P., Bessa R.J.B., Carolino I. (2020). A Comparison between the Quality of Eggs from Indigenous Chicken Breeds and That from Commercial Layers. Poult. Sci..

[20] Cendron F., Currò S., Rizzi C., Penasa M., Cassandro M. (2023). Egg Quality of Italian Local Chicken Breeds: II. Composition and Predictive Ability of VIS-Near-InfraRed Spectroscopy. Animals.

[21] Rizzi C. (2023). A Study on Egg Production and Quality According to the Age of Four Italian Chicken Dual-Purpose Purebred Hens Reared Outdoors. Animals.

[22] Sözcü A., İpek A., Oguz Z., Gunnarsson S., Riber A.B. (2021). Comparison of Performance, Egg Quality, and Yolk Fatty Acid Profile in Two Turkish Genotypes (Atak-S and Atabey) in a Free-Range System. Animals.

[23] Krawczyk J., Sokołowicz Z., Szymczyk B. (2011). Effect of Housing System on Cholesterol, Vitamin and Fatty Acid Content of Yolk and Physical Characteristics of Eggs from Polish Native Hens. Eur. Poult. Sci..

[24] Zemková Ľ., Simeonovová J., Lichovníková M., Somerlíková K. (2007). The Effects of Housing Systems and Age of Hens on the Weight and Cholesterol Concentration of the Egg. Czech J. Anim. Sci..

[25] Hargis P.S. (1988). Modifying Egg Yolk Cholesterol in the Domestic Fowl—A Review. World’s Poult. Sci. J..

[26] Horwitz W., Latimer G.W., AOAC INTERNATIONAL (2005). Official Method 925.30: Solids (Total) in Eggs—Vacuum Method. Official Methods of Analysis of AOAC INTERNATIONAL.

[27] Boehringer Mannheim, R-Biopharm (2017). Cholesterol—Colorimetric Method for the Determination of Cholesterol in Foodstuffs and Other Materials; Cat. No. 10 139 050 035. Instructions for Use. https://www.r-biopharm.com.

[28] ICH (2005). Q2(R1) Validation of Analytical Procedures: Text and Methodology.

[29] National Institute of Standards and Technology (NIST) (2020). Certificate of Analysis: Standard Reference Material 1845a—Whole Egg Powder.

[30] SAS Institute Inc. (2016). SAS/STAT^®^ 14.2 User’s Guide.

[31] Conover W.J., Iman R.L. (1981). Rank Transformations as a Bridge between Parametric and Nonparametric Statistics. Am. Stat..

[32] Tharrington J.B., Curtis P.A., Jones F.T., Anderson K.E. (1999). Comparison of Physical Quality and Composition of Eggs from Historic Strains of Single Comb White Leghorn Chickens. Poult. Sci..

[33] Lieboldt M.-A., Halle I., Frahm J., Schrader L., Baulain U., Henning M., Preisinger R., Weigend S., Dänicke S. (2015). Phylogenic versus Selection Effects on Growth Development, Egg Laying and Egg Quality in Purebred Laying Hens. Eur. Poult. Sci..

[34] Anene D.O., Akter Y., Thomson P.C., Groves P., Liu S., O’Shea C.J. (2021). Hens That Exhibit Poorer Feed Efficiency Produce Eggs with Lower Albumen Quality and Are Prone to Being Overweight. Animals.

[35] Sun C., Lu J., Yi G., Yuan J., Duan Z., Qu L., Xu G., Wang K., Yang N. (2015). Promising Loci and Genes for Yolk and Ovary Weight in Chickens Revealed by a Genome-Wide Association Study. PLoS ONE.

[36] Zhou L., Shi Y., Guo R., Liang M., Zhu X., Wang C. (2014). Digital Gene-Expression Profiling Analysis of the Cholesterol-Lowering Effects of Alfalfa Saponin Extract on Laying Hens. PLoS ONE.

[37] Vorlová L., Sieglová E., Karpíšková R., Kopřiva V. (2001). Cholesterol Content in Eggs during the Laying Period. Acta Vet. Brno.

[38] Rizzi C., Marangon A. (2012). Quality of Organic Eggs of Hybrid and Italian Breed Hens. Poult. Sci..

[39] Becker W.A., Spencer J.V., Verstrate J.A., Mirosh L.W. (1977). Genetic Analysis of Chicken Egg Yolk Cholesterol. Poult. Sci..

[40] Marks H.L., Washburn K.W. (1977). Divergent Selection for Yolk Cholesterol in Laying Hens. Br. Poult. Sci..

[41] Griffin H.D. (1992). Manipulation of Egg Yolk Cholesterol: A Physiologist’s View. World’s Poult. Sci. J..

[42] Yang P.K., Tian Y.D., Sun G.R., Jiang R.R., Han R.L., Kang X.T. (2013). Deposition Rule of Yolk Cholesterol in Two Different Breeds of Laying Hens. Genet. Mol. Res..

[43] Smith C.C., Fretwell S.D. (1974). The Optimal Balance between Size and Number of Offspring. Am. Nat..

[44] Martin T.E., Bassar R.D., Bassar S.K., Fontaine J.J., Lloyd P., Mathewson H.A., Niklison A.M., Chalfoun A. (2006). Life-History and Ecological Correlates of Geographic Variation in Egg and Clutch Mass among Passerine Species. Evolution.

[45] Ko E.-Y., Saini R.K., Keum Y.-S., An B.-K. (2021). Age of Laying Hens Significantly Influences the Content of Nutritionally Vital Lipophilic Compounds in Eggs. Foods.

[46] Zhang J., Gao X., Zheng W., Wang P., Duan Z., Xu G. (2023). Dynamic Changes in Egg Quality, Heritability and Correlation of These Traits and Yolk Nutrient throughout the Entire Laying Cycle. Foods.

[47] Mench J.A. (2002). Broiler Breeders: Feed Restriction and Welfare. World’s Poult. Sci. J..

[48] Dermane A., Eloh K., Palanga K.K., Adjito D.T., N’nanle O., Karou D.S., Kpanzou T.A., Caboni P. (2024). Comparative Metabolomic Profiling of Eggs from Three Diverse Chicken Breeds Using GC–MS Analysis. Poult. Sci..

[49] Guo H., Zhang X., You M., Shen Y., Zhang S., Li J., He X., Zhao X., Ma N. (2024). Quantitative Lipidomics Reveals the Changes of Lipids and Antioxidant Capacity in Egg Yolk from Laying Hens with Fatty Liver Hemorrhagic Syndrome. Poult. Sci..

[50] Meuwissen T.H.E., Hayes B.J., Goddard M.E. (2001). Prediction of Total Genetic Value Using Genome-Wide Dense Marker Maps. Genetics.

[51] Wolc A., Kranis A., Arango J., Settar P., Fulton J.E., O’Sullivan N.P., Avendano A., Watson K.A., Hickey J.M., de los Campos G. (2016). Implementation of Genomic Selection in the Poultry Industry. Anim. Front..

[52] Lourenco D., Legarra A., Tsuruta S., Masuda Y., Aguilar I., Misztal I. (2020). Single-Step Genomic Evaluations from Theory to Practice: Using SNP Chips and Sequence Data in BLUPF90. Genes.

[53] Greenfield H., Southgate D.A.T. (2003). Food Composition Data: Production, Management and Use.

[54] Finglas P.M., Berry R., Astley S. (2014). Assessing and Improving the Quality of Food Composition Databases for Nutrition and Health Applications in Europe: The Contribution of EuroFIR. Adv. Nutr..

