# Supplementary material for: Effects of Genetic and Production Type on Egg Cholesterol and the Yolk–Albumen Ratio in Slovenian Chicken Genotypes Under Standardised Conditions
Source: Animals (Basel). 2025 Dec 14;15(24):3588. doi: 10.3390/ani15243588 (PMC12729545; doi:10.3390/ani15243588)
Supplement: Supplementary file 1 [file animals-15-03588-s001.zip › animals-4016983-supplementary.pdf]

Supplementary Materials

to the article: *Effects of Genetic and Production Type on Egg Cholesterol and the Yolk–Albumen Ratio in Slovenian Chicken Genotypes under Standardised Conditions*

Authors: Dušan Terčič, Alenka Levart

Affiliation: University of Ljubljana, Biotechnical Faculty, Department of Animal Science, Ljubljana 1000, Slovenia

Corresponding author: dusan.tercic@bf.uni-lj.si; Tel: +386 1 320 3 915

The following supplementary tables provide additional data supporting the results presented in the main text.

Table S1. Nutrient composition, ingredient list, and additives of the complete corn–soybean meal–based diet.[1]

Parameter	Value / Description
Metabolizable energy (ME)	11.3 MJ kg^{-1}
Crude protein	16.2%
Crude fibre	3.0%
Crude fat	3.1%
Crude ash	12.5%
Lysine	0.65%
Methionine	0.25%
Calcium	3.60%
Phosphorus	0.20%
Sodium	0.75%
Ingredients	Corn (8.5% CP); Soybean meal (49.5% CP); Soybean oil; Sunflower meal (41.5% CP); Corn gluten meal (60% CP); Sugar beet molasses (10.1% CP); Calcium carbonate; Calcium–sodium phosphate; Monocalcium/dicalcium phosphate; Sodium bicarbonate; Sodium chloride; Feed additives.
Additives per kg of feed	10 000 IU vitamin A (E672); 3 000 IU vitamin D3 (E671); 25 mg iron (E1); 6 mg copper (E4); 100 mg manganese (E5); 60 mg zinc (E6); 0.3 mg cobalt (E3); 0.68 mg iodine (E2); 0.15 mg selenium (E8); 5.4 mg BHA (E320) and BHT (E321); 9.33 mg ethoxyquin (E324); 1.0 mg ethyl ester of β-apo-8-carotenoic acid (E160f); 4.0 mg canthaxanthin (E161g); 12.0 mg lutein (E161b); 50 mg 6-phytase (E1614(i)); 100 mg endo-1,4-β-glucanase (800 U/g).

[1]Nutrient values are derived from the official feed declaration provided by the manufacturer (Jata Emona d.o.o., Ljubljana, Slovenia). Ingredient inclusion rates are proprietary and therefore not disclosed.

Table S2. Average egg, albumen, and yolk masses, proportions, and yolk-to-albumen ratio in eggs from purebred and crossbred hens.

Purebred / Crossbred	Number of eggs sampled (n)	Average egg mass (g ± SD)	Average albumen mass (g ± SD)	Average yolk mass (g ± SD)	Albumen proportion (% ± SD)	Yolk proportion (% ± SD)	Yolk-to-albumen ratio (ratio ± SD)
SBH[1]	12	68.53 ± 2.84	40.57 ± 2.47	18.35 ± 0.84	59.21 ± 2.84	26.80 ± 1.51	0.45 ± 0.04
SSH[1]	12	70.41 ± 2.91	44.25 ± 2.86	17.77 ± 0.95	62.81 ± 1.95	25.27 ± 1.53	0.40 ± 0.04
SBaH[1]	12	65.79 ± 1.98	40.50 ± 1.84	18.56 ± 1.30	61.57 ± 2.12	28.20 ± 1.64	0.46 ± 0.04
SH[1]	12	54.85 ± 1.20	31.65 ± 0.78	17.23 ± 0.73	57.71 ± 0.93	31.40 ± 1.00	0.54 ± 0.02
Px-B[2]	12	73.87 ± 2.55	45.82 ± 2.02	19.35 ± 1.25	62.03 ± 1.58	26.20 ± 1.52	0.42 ± 0.03
Px-Ba[2]	12	72.52 ± 3.72	43.35 ± 3.12	20.66 ± 1.30	59.73 ± 1.82	28.50 ± 1.33	0.48 ± 0.03
Px-Bl[2]	12	72.32 ± 2.68	44.94 ± 2.82	18.90 ± 0.66	62.10 ± 1.82	26.17 ± 1.32	0.42 ± 0.03
SEFH[3]	12	68.66 ± 3.10	39.81 ± 2.84	20.93 ± 1.47	57.93 ± 2.12	30.50 ± 2.10	0.53 ± 0.05
SLFH[3]	12	67.76 ± 2.54	39.83 ± 1.91	20.33 ± 1.30	58.78 ± 1.65	30.00 ± 1.52	0.51 ± 0.04
SMH[3]	10	76.92 ± 2.66	47.00 ± 2.42	21.51 ± 0.81	61.08 ± 1.25	27.98 ± 1.18	0.46 ± 0.03

[1] Layer-type purebreds; [2] Layer-type crossbreds; [3] Meat-type purebreds.

Abbreviations of chicken genotypes: SBH – Slovenian Brown Hen; SSH – Slovenian Silver Hen; SBaH – Slovenian Barred Hen; SH – Styrian Hen; SEFH – Slovenian Early Feathering Hen; SLFH – Slovenian Late Feathering Hen; SMH – Slovenian Meat Hen; PxB – Prelux Brown; PxBa – Prelux Barred; PxBl – Prelux Black.

Table S3. Cholesterol traits (C_Egg, C_YF) in eggs of Slovenian chicken genotypes under standardised conditions (LS-means ± 95% CI)

A) Cholesterol Traits: Effect of production type

Trait	Layer type	Meat type	p-value
C_Egg (mg g^{-1})	3.59^{a} (3.51–3.68)	4.52^{b} (4.36–4.68)	<0.0001
C_YF (mg g^{-1})	12.80^{a} (12.54–13.07)	15.98^{b} (15.57–16.40)	<0.0001

B) Cholesterol Traits: Effect of genetic type nested within production type

Trait	Crossbred – Layer	Purebred – Layer	Purebred – Meat	p-value
C_Egg (mg g^{-1})	2.99^{a} (2.87–3.11)	4.20^{b} (4.06–4.33)	4.52^{c} (4.36–4.68)	<0.0001
C_YF (mg g^{-1})	11.14^{a} (10.74–11.54)	14.47^{b} (14.12–14.81)	15.98^{c} (15.57–16.40)	<0.0001

C) Cholesterol Traits: Effect of breed nested within the combination of production and genetic type

Breed	Production type	Genetic type	C_Egg (mg g^{-1}) ($p < 0.0001$)	C_YF (mg g^{-1}) ($p = 0.0236$)
PxB	Layer type	Crossbred	2.85^{a} (2.64–3.06)	10.87^{a} (10.17–11.56)
PxBa	Layer type	Crossbred	3.05^{a} (2.82–3.28)	11.17^{a} (10.47–11.86)
PxBl	Layer type	Crossbred	3.07^{a} (2.86–3.29)	11.38^{a} (10.69–12.08)
SBH	Layer type	Purebred	3.90^{bc} (3.68–4.12)	14.19^{b} (13.50–14.88)
SBaH	Layer type	Purebred	4.31^{cd} (4.09–4.52)	14.93^{bc} (14.24–15.63)
SH	Layer type	Purebred	4.85^{e} (4.60–5.10)	14.71^{bc} (14.01–15.40)
SSH	Layer type	Purebred	3.72^{b} (3.49–3.95)	14.03^{b} (13.34–14.73)
SEFH	Meat type	Purebred	4.64^{de} (4.41–4.88)	15.86^{cd} (15.16–16.55)
SLFH	Meat type	Purebred	4.44^{de} (4.22–4.66)	15.16^{bc} (14.47–15.86)
SMH	Meat type	Purebred	4.49^{cde} (4.22–4.76)	16.92^{d} (16.16–17.68)

Different superscript letters (a–e) indicate significant differences between production types (panel A), between genetic types nested within production types (panel B), and between breeds (panel C) ($p < 0.05$; Tukey–Kramer test).

Abbreviations of cholesterol traits: C_Egg – cholesterol in whole egg; C_YF – cholesterol in fresh yolk.

Abbreviations of chicken genotypes: PxB – Prelux Brown; PxBa – Prelux Barred; PxBl – Prelux Black; SBH – Slovenian Brown Hen; SBaH – Slovenian Barred Hen; SH – Styrian Hen; SSH – Slovenian Silver Hen; SEFH – Slovenian Early Feathering Hen; SLFH – Slovenian Late Feathering Hen; SMH – Slovenian Meat Hen.
